# Neural Correlates of Emotion Regulation in Patients with Schizophrenia and Non-Affected Siblings

**DOI:** 10.1371/journal.pone.0099667

**Published:** 2014-06-18

**Authors:** Lisette van der Meer, Marte Swart, Jorien van der Velde, Gerdina Pijnenborg, Durk Wiersma, Richard Bruggeman, André Aleman

**Affiliations:** 1 Department of Neuroscience, University Medical Center Groningen, Groningen, The Netherlands; 2 Department of Rehabilitation, Lentis Psychiatric Institute, Zuidlaren, The Netherlands; 3 Research Department, Lentis Psychiatric Institute, Groningen, The Netherlands; 4 Department of Clinical Psychology and Experimental Psychopathology, University of Groningen, Groningen, The Netherlands; 5 Department of Psychotic Disorders, GGZ Drenthe, Assen, The Netherlands; 6 Department of Psychiatry, University Medical Center Groningen, Groningen, The Netherlands; 7 Rob Giel Research Center, University Medical Center Groningen, Groningen, The Netherlands; Bellvitge Biomedical Research Institute-IDIBELL, Spain

## Abstract

**Background:**

Patients with schizophrenia often experience problems regulating their emotions. Non-affected relatives show similar difficulties, although to a lesser extent, and the neural basis of such difficulties remains to be elucidated. In the current paper we investigated whether schizophrenia patients, non-affected siblings and healthy controls (HC) exhibit differences in brain activation during emotion regulation.

**Methods:**

All subjects (n = 20 per group) performed an emotion regulation task while they were in an fMRI scanner. The task contained two experimental conditions for the down-regulation of emotions (reappraise and suppress), in which IAPS pictures were used to generate a negative affect. We also assessed whether the groups differed in emotion regulation strategies used in daily life by means of the emotion regulation questionnaire (ERQ).

**Results:**

Though the overall negative affect was higher for patients as well as for siblings compared to HC for all conditions, all groups reported decreased negative affect after both regulation conditions. Nonetheless, neuroimaging results showed hypoactivation relative to HC in VLPFC, insula, middle temporal gyrus, caudate and thalamus for patients when reappraising negative pictures. In siblings, the same pattern was evident as in patients, but only in cortical areas.

**Conclusions:**

Given that all groups performed similarly on the emotion regulation task, but differed in overall negative affect ratings and brain activation, our findings suggest reduced levels of emotion regulation processing in neural circuits in patients with schizophrenia. Notably, this also holds for siblings, albeit to a lesser extent, indicating that it may be part and parcel of a vulnerability for psychosis.

## Introduction

Schizophrenia is a severe and complex disorder, not only characterized by delusions and hallucinations, but also by abnormalities in the processing of emotions [1], [2]. Literature suggests that the ability to regulate emotional stimuli is of great importance for human adaptation [3]–[5]. Studies in patients with schizophrenia [1], [2], [6], [7] and their non-affected siblings [8] suggest a hampered ability to regulate emotions, which is reflected in the neural circuits of patients and relatives [1], [2], [9].

Emotion regulation refers to the conscious or unconscious process by which the emotional experience is manipulated and the subsequent expression of these emotions [10]. The strategy by which we regulate our emotions influences how we experience our emotions and is also indicative of our well being as well as how well we function at an interpersonal level [11]. Different emotion regulation strategies have been distinguished; *antecedent-focused strategies* and *response-focused strategies*. Antecedent-focused strategies modulate the emotion generation process in an early stage, before the actual response has taken place. Response-focused strategies, on the other hand, modulate the emotional response in a later stage, once the emotional response has been generated [10]. Two strategies commonly used in daily life lend themselves to experimental manipulation: reappraisal (antecedent-focused) and suppression (response-focused) [10]. In reappraisal the meaning of the events leading to an undesired emotional state is reinterpreted, such that the emotional impact is diminished. This results in an adjustment of the complete trajectory of the emotional response, leading to a diminished experiential, behavioral and emotional response. In contrast, suppression refers to the inhibition of emotion-expressive behavior. This results in little or no change in emotional experience and increased sympathetic activation of the cardiovascular system [10].

As the liability to schizophrenia is partly heritable [12], relatives have a higher risk of developing the disorder. More specifically, research indicates that chances of developing schizophrenia when a sibling suffers from the disorder, is up to ten times higher than the incidence of schizophrenia in the general population [13]. In addition, a relationship between genetic vulnerability and the expression of subclinical psychotic-like experiences [14] as well as personality profiles [15] have been demonstrated. Previous research has demonstrated similar but less severe brain abnormalities in healthy relatives of patients with schizophrenia [9]. Moreover, non-affected relatives of schizophrenia patients have shown impairments in emotional and cognitive processing, though less severe than patients [8], [12], [16], [17]. This suggests that even though these relatives have not developed schizophrenia they do share some phenotypes of the disorder. Therefore, a relevant question is whether non-affected relatives employ insufficient or inadequate emotion regulation strategies, since this may increase the daily stress level and subsequently the risk for exacerbation of psychosis. Studying the neural correlates of emotion regulation in HC, non-affected siblings and patients may help to identify traits that are presumably core parts of the vulnerability to schizophrenia. Since the siblings do not have the confounds of antipsychotic medication and chronicity of the illness, it makes these subjects particularly interesting to study. In this study we specifically investigated the neural mechanisms underlying emotion regulation in patients with schizophrenia and non-affected siblings in comparison with HC.

Studies investigating the neural basis of reappraisal, most importantly reported involvement of dorso- and ventrolateral prefrontal cortex (D & VLPFC), medial frontal areas, and parietal areas including the inferior parietal lobule (IPL) (see Diekhof et al. for a meta-analysis [18]). A few studies investigated the neural basis underlying both reappraisal and suppression [19]–[21]. Reappraising negative stimuli elicited increased activation in dorso- and ventrolateral PFC and dorsal anterior cingulate cortex (DACC) [19], [20] and diminished activation in the limbic areas, such as insula and amygdala in comparison to non regulation control conditions [19]. In contrast, the literature reports less consistent areas of activation for suppression. Both increased [20] and decreased [19] activation in prefrontal areas (medial prefrontal and inferior frontal regions) and increased activation in the supramarginal gyrus [21], insula [19], [21] and amygdala [19] compared to a non-regulation condition have been reported. Goldin and colleagues [19] suggested that the PFC is involved in the down-regulation of the amygdala during reappraisal, whereas the lack of PFC activation in suppression resulted in maintenance or even increase of activation in limbic areas, such as the amygdala and insula.

With regard to schizophrenia, published studies on the use of emotion regulation strategies suggest that patients with psychosis or schizophrenia are more likely to use suppression as an emotion regulation strategy [7] and less likely to use the reappraisal strategy compared to healthy controls [6]. In another study such a relationship could not be demonstrated [22]. There is a body of evidence suggesting emotional dysregulation as a core feature of schizophrenia [1], [2]. Nevertheless, we know of only one study in which the neural correlates underlying emotion regulation in patients with schizophrenia were investigated [23]. Morris and colleagues [23] demonstrated prefrontal hypoactivation during cognitive reappraisal in patients compared to healthy controls, suggesting that patients may experience difficulties reappraising negative events. No study to date has examined the neural mechanisms of emotion regulation in non-affected siblings. However, it has been demonstrated that both patients with schizophrenia and their non-affected siblings show structural and functional alterations in brain areas related to emotion regulation such as the dorsolateral PFC, the medial PFC, the ACC and amygdala [2], [12]. During cognitive tasks, many studies demonstrated that patients showed decreased brain activation in aforementioned areas as compared to control whereas for siblings both increased and decreased activation has been reported [24].

In the current study, we hypothesized that non-affected siblings would not differ from patients in the use of emotion regulation strategies (i.e. less reappraisal and more suppression). Furthermore, we specifically tested the hypothesis whether both schizophrenia patients and non-affected siblings would show aberrant frontal and amygdala activation during emotion regulation compared to healthy controls.

## Materials and Methods

### Ethics Statement

The study was approved by the local ethics committee (Medisch Ethische Toetsings Commissie, University Medical Center Groningen) and carried out in accordance with the latest version of the Declaration of Helsinki. All participants provided written informed consent after the procedure had been fully explained before participating. To ensure full capacity to consent, only patients in a stable clinical condition were included in the study. Patients with acute psychosis were excluded from participation.

### Participants

Twenty patients with a diagnosis of schizophrenia according to DSM IV-TR criteria, twenty non-psychotic siblings and twenty healthy controls (HC) participated in the study. Patients were selected from various psychiatric institutions in the Netherlands. The diagnosis of the patients was confirmed with a diagnostic interview, the Mini-International Neuropsychiatric Interview (MINI-Plus) [25]. All non-psychotic siblings participated in the GROUP (Genetic Risk & Outcome of Psychosis) study [26]. Siblings and controls were without a history of psychiatric illness, as assessed with either the Schedule for the Clinical Assessment in Neuropsychiatry (SCAN) [27] or the MINI-Plus. Participants were screened for MRI contra-indications, and drug- and medication use. Demographic and clinical characteristics are presented in table 1.

**Table 1:** Demographic information for the healthy controls, siblings and patients.

Demographic information	Controls (N = 20)	Siblings (N = 20)	Patients (N = 20)
Age (mean ± SD)^a^	35.5±11.7	32.6±8.6	35.2±10.8
Gender (n male)^b^	14	11	16
Level of education (mean ± SD)^b^	5.9±0.9	6.0±0.8	5.7±1.0
Age of Illness onset (mean ± SD)	n.a.	n.a.	24.7±8.1
PANSS positive (mean ± SD)	n.a.	n.a.	14.7±5.4
PANSS negative (mean ± SD)	n.a.	n.a.	14.7±4.1
PANSS general (mean ± SD)	n.a.	n.a.	29.9±7.7
Medication (n)	none	none	Aripirazole (7)
			Citalopram (3)
			Clomipranine (1)
			Clozapine (2)
			Fluoxetine (2)
			Haldol (1)
			Mirtazapine (1)
			Olanzapine (9) Oxazepam (3)
			Paroxetine (1)
			Perfenazine (1)
			Quetiapine (3)
			Risperidone (1)
			Temazepam (1)
			Venlafaxine (1)

a Groups were compared with an analysis of variance (ANOVA) and did not differ significantly (p = 0.62).

b Level of education was defined according to scoring system of Verhage [57] (ordinal scale). Group differences for gender and education were tested with a chi-square and kruskal-wallis test, respectively. Neither gender (p = 0.23) nor level of education (p = 0.72) differed between groups.

### Tasks and Questionnaires

#### Diagnosis and clinical characteristics

The Mini-International Neuropsychiatric Interview - Plus [25] and the Schedule for the Clinical Assessment in Neuropsychiatry (SCAN) [27] are structured diagnostic interviews. These were used to confirm the diagnosis made by the psychiatrist, according to the DSM-IV and ICD-10 criteria and to assess psychiatric history of siblings and healthy controls. In addition, to assess the clinical characteristics in the past week, the semi-structured interview Positive and Negative Syndrome Scale (PANSS) [28] was administered in all patients by a trained interviewer. The PANSS consists of three subscales: positive symptoms (P), negative symptoms (N), and general psychopathology (G) and has good inter-rater reliability and validity [29].

#### Emotion regulation questionnaire

The self-reported use of the emotion regulation strategies reappraisal and suppression people apply in every day life, was assessed with the Dutch translation of the Emotion Regulation Questionnaire (ERQ) [11]. The ERQ consists of ten items, four items measuring suppression (e.g. “I keep my emotions to myself”) and six items measuring reappraisal (e.g. “When I want to feel more positive emotion, I change the way I’m thinking about the situation”). Subjects rated on a 7-point scale (strongly disagree - strongly agree) to what extent the statements applied to them. The ERQ is a reliable measure of emotion regulation (Cronbach’s alpha of 0.79 for reappraisal and 0.73 for suppression [11]). To assess the relationship between both strategies, total scores of both subscales were divided by the number of items per subscale to calculate the score per subscale.

#### Emotion regulation task

**Figure 1:**
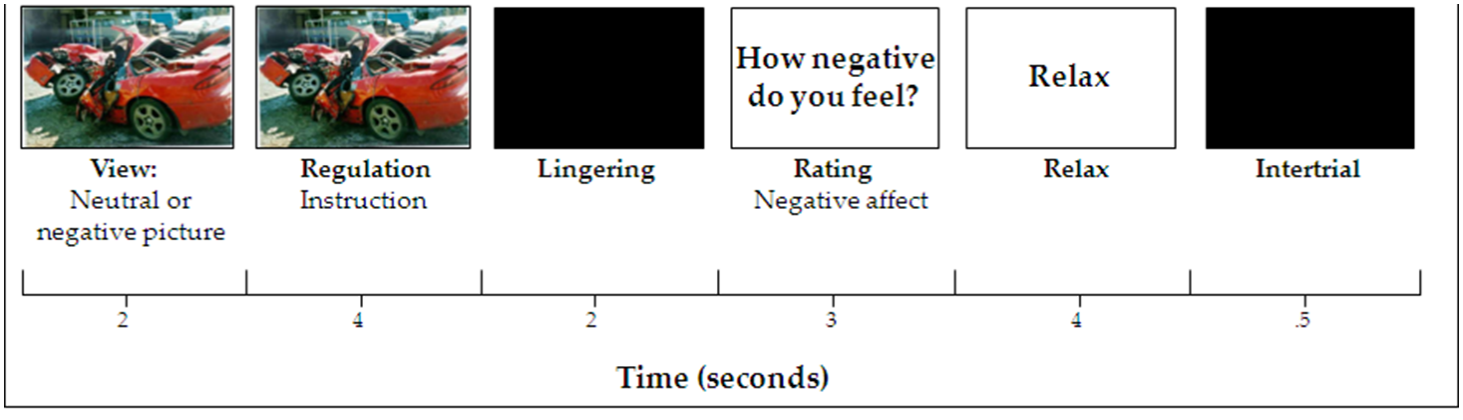
Experimental design for a single trial. The task consisted of 110 trials (22 neutral and 88 negative). A single trial lasted 15,5 seconds. Every 9 or 10 trials were followed by a fixation cross of 20 seconds.

The emotion regulation task as developed by Ochsner [30] is based on the theoretical framework of Gross [10]. This task has been adopted by several studies to measure the neural correlates of emotion regulation and demonstrated a consistent pattern of activation in the reappraisal condition [16], [17], [22], [29], [49]. At the time of designing the current experiment, only one paper had published neuroimaging results with regard to the suppression condition [19]. Therefore, we included a suppression condition designed in a similar way as the reappraisal condition and similar instructions as were used by Goldin et al. [19]. The task was presented with E-Prime (Psychology Software Tools Inc, Pittsburg). Stimuli were emotional pictures, extracted from the International Affective Picture System (IAPS). The task consisted of four main conditions: attend neutral, attend negative, reappraise and suppress, each containing 22 trials. Stimulus selection was randomized within negative and neutral conditions (mean valence negative = 2.6; mean arousal negative = 5.7; mean valence neutral = 1.3; mean arousal neutral = 1.9; see table S1 for an overview of included pictures). Each trial (see figure 1) was constructed as follows. First, a picture was presented for two seconds with the instructions just to ‘look’ at the picture (View). Subsequently, while the picture remained on the screen, a written instruction appeared below the picture. This instruction was presented on the screen for a duration of 4 seconds and indicated how the subject was supposed to regulate (reappraise, suppress or attend) the emotion triggered by the picture (Regulation). Instructions were in accordance with Ochsner et al. [30] and Goldin et al. [19]. When asked to reappraise, subjects had to reinterpret the picture in such a way that its negativity decreased, making it less emotionally disturbing (e.g. a picture of a severe car accident then becomes a car accident in which only superficial damage was done and no one was injured). The instruction “suppress” required the subject to suppress the emotion elicited by the picture. This was illustrated by giving the example that someone else should not be able to read the emotion on the subjects’ face (i.e. keeping a poker face). Instructions for ‘attend’ were simply to look at the picture and not change the emotions they were feeling. This instruction was the same for the attend neutral condition and the attend negative condition (thus, since the picture remained on the screen and the instructions for both “attend” and “view” were to look at the picture and experience the elicited emotion, “attend” is simply a continuation of “view” in both the attend neutral and attend negative conditions). Following the regulation, a black screen was presented for two seconds to let the emotions linger (Lingering). Then, subjects were asked to rate how negative they felt at the moment of rating (three seconds; Rating). Finally, the word ‘relax’ was presented for four seconds where subjects could relax (Relax), followed by a black screen (0.5 seconds) to alert participants that the next trial was coming (Intertrial interval). Each trial had a duration of 15.5 seconds. Fourteen additional rest blocks were included, one restblock followed every tenth trial, in which a fixation cross was presented for 20 seconds (Fixation).

Prior to the fMRI scan each subject received training to ensure a complete understanding of the task. During this training the subjects practiced the different strategies on negative pictures by telling the researcher how they would apply the strategy, until they understood the task. Pictures used in the training were not used for the experimental task. In case of incomplete understanding of the task, the training was repeated.

The schizophrenia patients and siblings were scanned for two separate studies. For the former group, an additional condition was included: Increase. Instead of down-regulating their negative affect, subjects had to increase the negative affect that was induced by the stimulus. Since this condition was not administered in the siblings, it could not be included in the analysis of the current study. Whether this may influence the results, we checked in the healthy control group. Of the healthy controls (HC) 12 subjects participated in the study including the increase condition and 8 not including the increase condition. Comparing the mean rating of these HC subjects did not reveal a significant difference in the rating of negative affect. We therefore conclude that this will not influence the results.

### Data Acquisition

FMRI data was acquired using a 3.0 Tesla whole body scanner equipped with an 8 channel SENSE head coil (Philips Intera, Best, NL). The functional images were acquired by a T2-weighted echo producing 37 slices of 3.5 mm thick with no gap and the images were slightly tilted (30 degrees) to prevent artifacts due to the nasal cavities. The functional scans were made in the axial plane (TR = 2 s; TE = 30 s; flip angle (α) 70°; FOV = 224.0, 129.5, 224.0; in-plane resolution 64×62 pixels; isotropic voxels of 3.5 mm) and were scanned interleaved. For anatomical reference, a T1-weighted image (170 slices; isotropic voxels of 1 mm; TR 9 ms; TE 3.54 ms; α 8°; FOV 256 mm) was acquired in the bicommissural plane, covering the whole brain.

### Statistical Analyses

#### Behavioral analyses

The behavioral data was analyzed using SPSS 16 (SPSS Inc., Chicago, IL, USA). Two separate ANOVAs were performed for the Reappraisal and Suppression subscales of the ERQ with Group (patients, siblings, HC) as an independent variable.

For the emotion regulation task, the degree of negative affect (rating) and the reaction times (RT) of the rating were analyzed with two repeated measures ANOVAs: one for rating and one for RTs with Condition (attend neutral, attend negative, reappraise and suppress) as within-subject variables and Group as between-subject factor. For all the statistical analyses significance level was set at p<.05 two-tailed.

#### fMRI analyses

The fMRI data were analyzed with Statistical Parametric Mapping (SPM 5) (www.fil.ion.ucl.ac.uk) in Matlab7 (The MathWorks Inc., Natick, MA, USA). Orientation of the functional images was adjusted by hand, based on the anatomical image. Subsequently, data were preprocessed by applying slice timing correction, realignment and coregistration. Coregistrations were controlled manually for each subject to ensure correct coregistration. Functional images were spatially normalized on the basis of the MNI (Montreal Neurological Image) T1 template. Finally, the images were smoothed with a 3D isotropic 10 mm full-width/half-maximum (FWHM) Gaussian Kernel.

At first level, sixteen regressors were modeled with a boxcar function convolving a hemodynamic response function. The regressors View and Relax were subdivided into Neutral and Negative, while the regressors Condition, Linger and Rating were subdivided into reappraise, suppress, attend negative and attend neutral. Fixation and intertrial interval together formed the baseline brain activation. For each participant, five contrasts were defined 1) view negative versus view neutral 2) attend neutral versus fixation 3) attend negative versus fixation 4) reappraise versus fixation and 5) suppress versus fixation.

First, the contrast images of attend negative vs fixation, reappraise vs fixation and suppress vs fixation were entered into a full-factorial model (3×3 ANOVA) with Group (HC, siblings, patients) and Condition (attend negative, reappraise, suppress) as factors. To validate the task by confirming involvement of the PFC during regulation, activation for the regulation conditions were examined in healthy controls only with the contrasts (reappraisal>attend negative) and (suppression>attend negative). All analyses for healthy controls only were thresholded at p<0.001, k≥20 and FWE-cluster level corrected at p<0.05. Subsequently, between group differences were examined with the T-contrasts (reappraisal>attend negative) and (suppression>attend negative). For between group differences a more liberal threshold of p<0.001 and k≥20 was used to prevent type II errors.

## Results

### Behavioral Data

Demographic data are presented in table 1. ERQ scores and ratings for the emotion regulation task are presented in table 2. Emotion regulation ratings for one healthy control was not available due to technical errors. For the other analyses, these data were included since brain activation was not different from other HC.

**Table 2:** Mean scores on questionnaires and on the emotion regulation task for healthy controls, siblings and patients.

	Controls (N = 19)^a^	Siblings (N = 20)	Patients (N = 20)
	Mean ± SD	Mean ± SD	Mean ± SD
Tasks and questionnaires			
ERQ			
Reappraisal	5.0±1.0	4.6±1.0	4.4±1.3
Suppression	2.9±1.2	3.1±.09	3.5±1.1
Rating Emotion Regulation			
Attend negative	2.3±0.5	2.6±0.6	2.7±0.6
Reappraise	1.9±0.6	2.2±0.5	2.4±0.7
Suppress	2.1±0.6	2.5±0.6	2.4±0.6
Attend neutral	1.1±0.1	1.1±0.1	1.3±0.2

a For one subject, no behavioral data for the emotion regulation task were available due to technical problems.

#### ERQ

With regard to ERQ ratings, no significant group differences were found for neither the reappraisal nor the suppression subscale (see table 2 for mean scores).

#### Emotion regulation task

**Figure 2:**
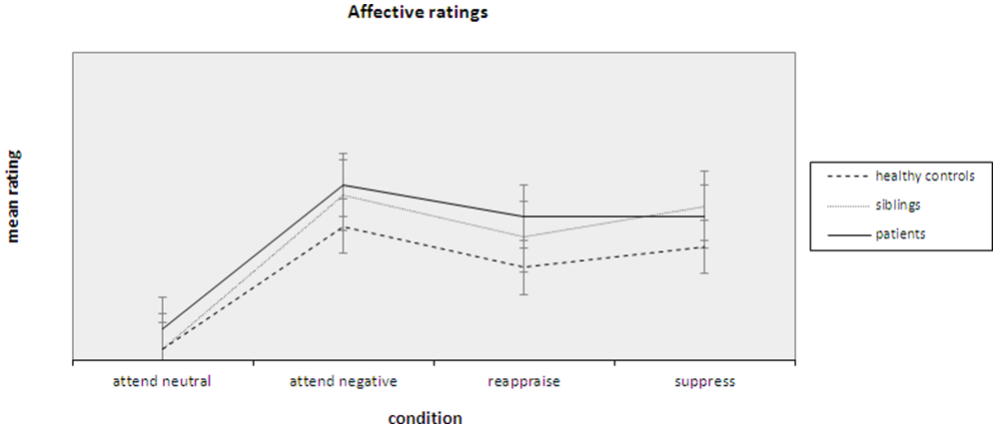
Affective ratings for the emotion regulation task per condition. Lines represent the three groups separately.

Ratings for the emotion regulation task are presented in table 2 and visualized in figure 2. To ensure that no a priori differences in negative affect were present that may have influenced affect ratings for the emotion regulation task, group differences for Positive Affect and Negative Affect Scale (PANAS [31]) scores were assessed. An ANOVA demonstrated no significant group differences for positive affect (PA; p = 0.48) nor for negative affect (NA; p = 0.17). A significant main effect was found for Condition (attend neutral, attend negative, reappraise and suppress) on emotion ratings during the task [F (3,54) = 122.48, p<0.0001]. Pairwise comparisons demonstrated that ratings for all conditions were significantly different (maximum p = 0.019), which confirms the induction of negative affect by the presented stimuli. Furthermore, a main effect for Group (patients, siblings and HC) was found [F (2,56) = 4.95, p = 0.010]. Pairwise comparisons showed that patients did not differ significantly from siblings (p = 0.369), but rated stimuli significantly more negative than HC (p = 0.003). Similarly, siblings rated stimuli significantly more negative than HC (p = 0.034). Finally, no Group x Condition interaction could be demonstrated [F (6,110) = 1.19, p = 0.315]. Thus, while patients and siblings showed significantly higher negative ratings than HC, all groups seemed to have been able to regulate their negative affect with both the reappraisal and the suppression strategy.

### Imaging Results

#### Main task effect

**Figure 3:**
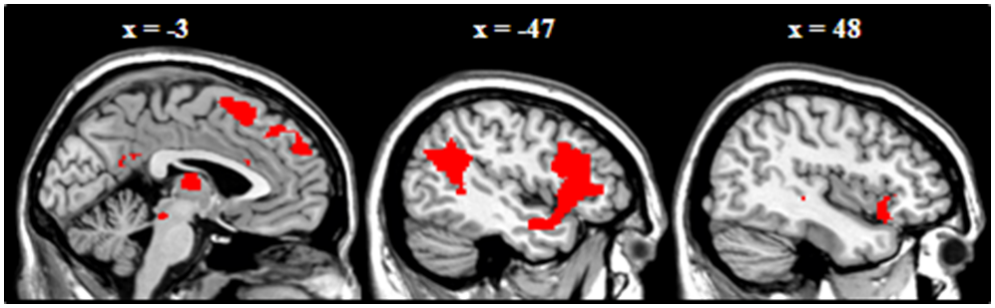
Activation for HC only during reappraisal.

To confirm whether the task would index prefrontal involvement in emotion regulation areas as previously reported in the literature [19], [30], we examined activation patterns in HC for the contrasts (reappraise>attend neg) and (suppress>attend negative). Most importantly, the contrast (reappraise>attend negative) yielded bilateral activation in the dorsolateral prefrontal cortex (DLPFC), anterior insula and superior & middle temporal gyrus (S&MTG), the dorsomedial prefrontal cortex [DMPFC; including superior frontal gyrus (SFG)], the right ventrolateral prefrontal cortex (VLPFC), the left supramarginal gyrus (SMG), and left inferior parietal lobe (IPL) (figure 3; table 3). The reverse contrast (attend negative>reappraise) did not yield any activation. Finally, for the contrast (suppress>attend negative) no areas of activation could be demonstrated; the inverse contrast (attend negative>suppress) did not yield any relevant clusters of activation either (see table 4).

**Table 3:** fMRI results for the contrast Reappraise >Attend negative.

					MNI coordinates		
Region of activation	L/R	N voxels	T	Z	x	y	z
*Healthy controls only (FWE clustercorrected, p<0.05,)*							
Inferior frontal gyrus (V & DLPFC)	L	2273	7.23	6.75	−38	24	−8
			6.63	6.24	−32	28	−4
			5.95	5.67	−50	24	8
Inferior frontal gyrus/insula	R	135	4.57	4.43	42	20	−12
Superior frontal gyrus (DMPFC)	L	519	5.85	5.58	−6	16	62
		370	5.03	4.85	−6	56	34
			4.49	4.36	−10	42	48
			3.45	3.39	−2	38	44
Superior & Middle Temporal Gyrus (STG/MTG)	L	1103	4.68	4.53	−42	−54	22
			4.39	4.26	−38	−46	22
			4.31	4.19	−52	−50	4
	R	81	4.2	4.09	58	−4	−8
			3.83	3.74	56	6	−20
Parahippocampal gyrus	L	327	4.36	4.24	−10	−34	−2
			4	3.91	−6	−30	−10
Thalamus	R	152	4.24	4.13	0	−14	10
			3.19	3.14	10	−8	4
Caudate	L	58	3.87	3.78	−12	4	12
	R	57	4.24	4.13	12	16	6
Posterior cingulate gyrus	L	32	3.5	3.44	−4	−48	28
			3.27	3.22	−2	−56	22
*Healthy controls>schizophrenia patients (p<0.001. unc.)*							
Inferior frontal gyrus/insula	L	24	3.66	3.59	−38	22	−10
Middle temporal gyrus (MTG)	L	37	3.95	3.86	−38	−66	26
Parahippocampal gyrus	L	44	3.98	3.89	−12	−34	0
Caudate	R	31	4.14	4.03	8	20	6
Thalamus	R	24	3.51	3.44	2	−14	8
*Healthy controls>siblings (p<0.001. unc.)*							
Superior temporal gyrus (STG)	L	109	4.22	4.11	−44	8	−18
Inferior frontal gyrus (IFG)			3.7	3.62	−40	18	−14
Parahippocampal gyrus/amygdala	L	96	3.84	3.76	−16	−4	−18
			3.5	3.43	−22	2	−18
			3.49	3.42	−18	−14	−20
	L	51	3.69	3.62	−10	−34	−2
*Siblings>healthy controls (p<0.001. unc.)*							
Middle frontal gyrus	R	20	3.42	3.36	42	4	40
*Siblings>Schizophrenia patients (p<0.001. unc.)*							
Inferior parietal lobe (IPL)	L	66	4.23	4.12	−38	−70	28
Caudate	L	41	3.56	3.49	−14	14	4
			3.47	3.4	−6	8	2
	R	23	3.93	3.84	12	18	6

L, Left; R, Right; MNI, Montreal Neurological Institute; ACC, anterior cingulate cortex; DLPFC, dorsolateral prefrontal cortex; DMPFC, dorsomedial prefrontal cortex; VLPFC, ventrolateral prefrontal cortex.

**Table 4:** fMRI results for the contrast Attend negative>Suppress.

					MNI coordinates		
Region of activation	L/R	N voxels	T	Z	x	y	z
*Healthy controls only (FWE clustercorrected, p<0.05,)*							
Lingual gyrus	L	190	4.01	3.91	−16	−70	4
			3.60	3.53	−14	−78	4
Middle Occipital gyrus	L	229	3.86	3.78	−36	−74	30
			3.79	3.71	−30	−66	38
			3.40	3.34	−20	−64	48

L, Left; R, Right; MNI, Montreal Neurological Institute.

#### Group differences

**Figure 4:**
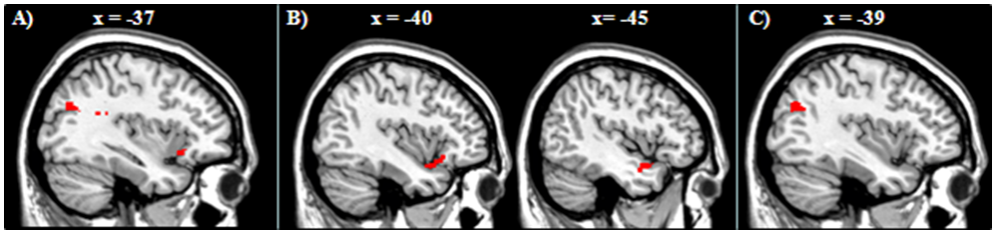
Group comparisons during reappraisal. A) HC versus schizophrenia patients. B) HC versus non affected siblings. C) Non-affected siblings versus schizophrenia patients.

With regard to group differences, we first tested differences between HC and patients, since we had explicit expectations concerning this comparison. For the contrast (reappraise>attend negative) HC showed more activation in the left VLPFC, including the left anterior insula (figure 4A; table 3). The reverse comparison, patients versus HC, did not reveal any differences. Comparing HC with siblings revealed decreased activation for siblings in the left VLPFC, STG and amygdala (figure 4B; table 3). The inverse contrast (siblings>HC) did not reveal any relevant differences in activation. Finally, siblings showed more activation in the left IPL than patients (figure 4C; table 3). Patients did not show areas of higher activation compared to siblings.

## Discussion

This study is the first to investigate the neural basis of emotion regulation in schizophrenia patients and siblings. Most importantly, we demonstrated decreased activation in the left VLPFC in both patients and siblings compared to HC, during reappraisal of emotion-inducing stimuli. All groups reported less negative affect after using either reappraisal or suppression. Groups did not differ in their reports of using either the reappraisal or suppression strategy in daily life, which is consistent with findings from Henry et al. [22]. However, this is inconsistent with earlier studies demonstrating an increase in the self-reported use of suppression for patients compared to HC [6], [7]. This needs further clarification in larger samples, as it may be related to heterogeneity in symptom profiles. That is, it has been suggested that different pathways towards psychotic disorders can be distinguished involved in psychotic disorders, with either more emphasis on affective dysregulation or on cognitive impairment [32].

### Emotion Regulation and the Brain

In all groups reappraising negative stimuli activated similar areas as reported in previous studies, namely DLPFC and VLPFC bilaterally, the DMPFC and ACC, left SFG and IPL [18], [19], [30], [33]. However, no activation was demonstrated in HC when suppressing negatively valenced stimuli. We suspect that this may have been due to the expressive suppression condition itself. Whether or not the subjects correctly applied this strategy (and not for example reappraised the negative stimulus) was not verifiable and thus the activation yielded by this condition was not reliable. Therefore, we decided not to test or discuss possible group differences for this condition. Goldin et al. [19] demonstrated that down-regulation of amygdala activation occurred relatively late in time (after 10–15 sec of the regulation instructions), which may explain why in the current analyses, which included the whole time phrame, no differences in amygdala activation could be demonstrated. In addition, our findings are consistent with a recent meta-analysis that showed that only 17 of 31 studies found hypoactivation of the amygdala during emotion regulation [18].

### Emotion Regulation in Schizophrenia Patients and Siblings

Behaviorally, both schizophrenia patients and siblings reported a decrease in their negative affect using reappraisal. However, both patients and siblings reported a greater overall negative affect over all conditions (attend neutral, attend negative, reappraisal & suppression) compared to HC. These findings are consistent with previous proposals [2], [34], [35] and imply that both patients and siblings show an elevated level of emotional reactivity compared to healthy subjects. Since emotional reactivity has been suggested to reflect the ability to deal with daily life stress [36]–[39], our results may reflect a lesser ability for both patients and siblings in this respect.

Despite their ability to reappraise negative stimuli, schizophrenia patients as well as siblings demonstrated less activation in the VLPFC (including the IFG, extending to the anterior insula) compared to HC during reappraisal. According to Ochsner [3] the VLPFC is mostly important in the context evaluation of emotional stimuli and the selection of subsequent actions, while the DLPFC is involved in more explicit processes such as reasoning and describing with regard to the changing of the emotional respons. Previous studies indicate that the VLPFC is involved in mentalizing abilities [40], more specifically inhibitory control processes underlying mentalizing [41], [42] and has strong anatomical connections with ventrolimbic areas central to emotional processing [43]. In addition, the anterior insula has been related to emotional processing and emotional awareness [44]. Hypoactivation in these areas has been related to compromised cognitive control and emotion regulation in patients with schizophrenia [23], [45]. Wylie and Tregallas [46] demonstrated decreased anterior insula activation in the processing of emotion stimuli in schizophrenia patients, which is consistent with the current findings. Leitman et al. [47] provided evidence for impaired VLPFC functioning in schizophrenia patients during facial affect appraisal. Finally, Stip et al. [48] showed a relationship between blunted affect in patients with schizophrenia and VLPFC functioning during emotion processing. Together, these findings suggest that even though all groups reported a decrease in negative affect after emotion regulation, our brain activation data do point towards group differences in the neural mechanisms underlying emotion regulation.

In relatives, only a few studies have investigated the neural substrates underlying emotional processing, with some studies reporting hyperactivity in limbic and medial frontal brain areas [49] and other studies reporting hypoactivity in limbic and lateral fontal brain areas [50], [51]. Similarly, studies investigating emotional processing in clinical high risk individuals showed varying results [52], [53]. Gee et al. [52] investigated emotional processing in clinical high risk individuals and concluded that the VLPFC seems to play an important role in the development of emotional processing. Modinos et al. [53] instead found increased activation in subjects prone to psychosis (but without clinical symptoms) in emotion regulation areas. Studies investigating cognitive processes also reported increased as well as decreased brain activation in high risk individuals, while for first episode patients mostly decreased activation is reported in frontal areas compared to healthy controls [24], [53].

The current behavioral findings seem to suggest that both patients and siblings are able to decrease their negative affect by adopting regulation strategies, despite diminished brain activation in the VLPFC. It is possible that both patients and siblings can indeed employ the reappraisal strategy in a laboratory setting, which is likely to be more structured and less complex than situations encountered in daily life. Barbalat et al. [54] demonstrated that an increase of complex contextual information was related to reduced task performance in patients with schizophrenia. Interestingly, an increase of complex contextual information was associated with hypoactivation in the left VLPFC in patients compared to HC. This is in line with the function of the VLPFC in emotion regulation as it was proposed by Ochsner and Gross [3], namely to evaluate the emotional context. Also, Gibson et al. [55] investigated complex social skills in high risk individuals who had no difficulties in understanding the beliefs and intentions of others (Theory of Mind). These individuals did show impairments in a more complex social task in which they were asked to audition for a new reality show (High-Risk Social Challenges task). Thus, increasing the number of social cues and a higher level of social interaction seems to be more difficult for high risk individuals. With regard to our results, we hypothesize that patients may have been able to reappraise their negative affect, because of the structured laboratory setting and the thorough task instructions. Should the complexity of the contextual information increase (eg. reappraising a negative event in daily life), patients may not be able to fully down-regulate their negative affect without clear instructions and a structured environment.

The finding that the performance of the siblings mostly resembles patients’ performance in both behavioral and functional results, suggests that the regulation of negative emotions may be a vulnerability marker for the development of pathological symptoms. Inadequate regulation of negative emotional events may make at risk individuals more prone to daily stress and increases risk for exacerbation of psychosis. This is supported by the elevated emotional reactivity levels for both patients and siblings that were found in this study, which has been suggested to reflect a decreased ability to deal with daily life stress [36]–[39]. However, more research is needed to investigate to what extent the complexity of contextual information is of influence on emotion regulation performance and how this knowledge can be used in a clinical setting. If indeed succesful emotion regulation depends upon the ability to evaluate complex social and environmental information, this may be a target for therapy.

### Limitations

All included subjects reported to be able to reappraise and suppress. For reappraisal the subjects received training prior to scanning, until they completely understood the task and were able to give an alternative interpretation for the presented picture. As discussed above, the ability to suppress was hard to verify, which made the suppression condition unreliable for interpretation. To investigate the strategy of expressive suppression, future studies will have to develop a valid task condition that yields reliable activation. To our best knowledge, no study to date has developed such a valid task condition. Furthermore, while healthy controls and siblings were medication free, patients did use medication. It is possible that this could have influenced activation patterns in patients. However, in a study by Sergi and colleagues no evidence for effects of antipsychotic medication on social cognitive performance could be demonstrated [56]. Finally, patients and siblings were scanned for two separate studies; the study examining emotion regulation in patients included one additional condition (Increase). However, as we mentioned in the methods section, whether or not this may have influenced the results was checked in the HC group. Since there were no differences in the rating of negative affect between the HC subjects from both studies, we concluded that this was not the case.

## Conclusions

Despite the fact that all groups reported decreased negative affect after the prompt to reappraise, patients and siblings showed higher negative affect ratings compared to healthy controls in all conditions. Both patients and siblings showed decreased activation in the left VLPFC during reappraisal of negative emotional stimuli compared to healthy controls. Possibly, the structured laboratory setting with thorough task instructions enabled patients and siblings to down-regulate. Difficulties in recruiting the prefrontal cortex for affect regulation may be a vulnerability marker for the development of pathological symptoms. However, more research is needed to investigate to what extent the complexity of contextual information is of influence on emotion regulation performance.

## Supporting Information

Table S1 **Selected IAPS pictures: mean valence and mean arousal ratings per stimulus.**
(DOCX)
