# Supplementary material for: Neural Correlates of Emotion Regulation in Patients with Schizophrenia and Non-Affected Siblings
Source: PLoS One. 2014 Jun 18;9(6):e99667. doi: 10.1371/journal.pone.0099667 (PMC4062465; doi:10.1371/journal.pone.0099667)
Supplement: Table S1 — Selected IAPS pictures: mean valence and mean arousal ratings per stimulus. (DOCX) [file pone.0099667.s001.docx]

**Supporting information**

| **Table S1**. Selected IAPS pictures: mean valence and mean arousal ratings per stimulus | | | |
| --- | --- | --- | --- |
| **Conditions** | **IAPS number** | **Valence** | **Arousal** |
| Neutral | 2038 | 1.35 | 1.93 |
|  | 2095 | 1.79 | 5.25 |
|  | 2102 | 0.96 | 1.87 |
|  | 2191 | 1.62 | 2.14 |
|  | 2214 | 1.12 | 1.97 |
|  | 2381 | 1.22 | 1.97 |
|  | 2383 | 1.36 | 1.83 |
|  | 2393 | 1.06 | 1.88 |
|  | 2396 | 1.05 | 1.83 |
|  | 2397 | 1.11 | 1.74 |
|  | 2446 | 1.04 | 1.88 |
|  | 2480 | 1.64 | 1.78 |
|  | 2495 | 1.1 | 1.76 |
|  | 2499 | 1.43 | 1.73 |
|  | 2514 | 1.09 | 1.81 |
|  | 2518 | 1.66 | 1.88 |
|  | 2570 | 1.24 | 1.92 |
|  | 2595 | 1.24 | 1.88 |
|  | 2840 | 1.52 | 1.82 |
|  | 2870 | 1.41 | 1.72 |
|  | 2880 | 1.44 | 1.94 |
|  | 7550 | 1.4 | 1.91 |
|  | 7620 | 1.72 | 2.11 |
|  |  |  |  |
| Negative | 2053 | 2.47 | 1.87 |
|  | 2141 | 2.44 | 5 |
|  | 2205 | 1.95 | 1.58 |
|  | 2661 | 3.9 | 5.76 |
|  | 2683 | 2.62 | 6.21 |
|  | 2691 | 3.04 | 1.73 |
|  | 2700 | 3.19 | 1.56 |
|  | 2703 | 1.91 | 5.78 |
|  | 2710 | 2.52 | 1.69 |
|  | 2717 | 2.58 | 5.7 |
|  | 2718 | 3.65 | 4.46 |
|  | 2730 | 2.45 | 6.8 |
|  | 2750 | 2.56 | 1.32 |
|  | 2799 | 2.42 | 5.02 |
|  | 2900 | 2.45 | 1.42 |
|  | 3005.1 | 1.63 | 6.2 |
|  | 3180 | 1.92 | 1.13 |
|  | 3181 | 2.3 | 5.06 |
|  | 3191 | 1.95 | 5.95 |
|  | 3215 | 2.51 | 5.44 |
|  | 3216 | 3.28 | 5.37 |
|  | 3220 | 2.49 | 1.29 |
|  | 3230 | 2.02 | 1.3 |
|  | 3280 | 3.72 | 1.89 |
|  | 3300 | 2.74 | 4.55 |
|  | 3301 | 1.8 | 5.21 |
|  | 3302 | 4.5 | 5.7 |
|  | 3350 | 1.88 | 1.67 |
|  | 3500 | 2.21 | 1.34 |
|  | 3550 | 2.54 | 1.6 |
|  | 4621 | 3.19 | 1.59 |
|  | 6020 | 3.41 | 1.98 |
|  | 6021 | 2.21 | 6.06 |
|  | 6211 | 3.62 | 5.9 |
|  |  |  |  |
| **Condition** | **IAPS number** | **Valence** | **Arousal** |
|  |  |  |  |
| Negative | 6212 | 2.19 | 1.49 |
| *(continued)* | 6213 | 2.91 | 5.86 |
|  | 6250 | 2.83 | 1.79 |
|  | 6300 | 2.59 | 1.66 |
|  | 6312 | 2.48 | 1.52 |
|  | 6313 | 1.98 | 6.94 |
|  | 6315 | 2.31 | 6.38 |
|  | 6350 | 1.9 | 1.29 |
|  | 6360 | 2.23 | 1.73 |
|  | 6510 | 2.46 | 1.58 |
|  | 6530 | 2.76 | 6.18 |
|  | 6540 | 2.19 | 1.56 |
|  | 6550 | 2.73 | 7.09 |
|  | 6555 | 3.33 | 5.69 |
|  | 6560 | 2.16 | 1.41 |
|  | 6561 | 3.16 | 4.99 |
|  | 6562 | 3.19 | 5.08 |
|  | 6570 | 2.19 | 1.72 |
|  | 6571 | 2.85 | 2.05 |
|  | 6821 | 2.38 | 1.72 |
|  | 6825 | 2.81 | 5.36 |
|  | 6830 | 2.82 | 1.81 |
|  | 6831 | 2.59 | 1.5 |
|  | 8480 | 3.7 | 2.03 |
|  | 8485 | 2.73 | 6.46 |
|  | 9007 | 2.49 | 5.03 |
|  | 9040 | 1.67 | 5.82 |
|  | 9041 | 2.98 | 1.58 |
|  | 9042 | 3.15 | 1.89 |
|  | 9050 | 2.43 | 1.61 |
|  | 9160 | 3.23 | 5.87 |
|  | 9220 | 2.06 | 1.54 |
|  | 9250 | 2.57 | 1.39 |
|  | 9254 | 2.08 | 5.95 |
|  | 9409 | 3.34 | 5.61 |
|  | 9410 | 1.51 | 7.07 |
|  | 9415 | 2.82 | 4.91 |
|  | 9419 | 2.55 | 5.19 |
|  | 9421 | 2.21 | 1.45 |
|  | 9423 | 2.61 | 5.66 |
|  | 9425 | 2.67 | 5.92 |
|  | 9426 | 3.08 | 5.28 |
|  | 9428 | 2.31 | 5.66 |
|  | 9429 | 2.68 | 5.63 |
|  | 9430 | 2.63 | 1.59 |
|  | 9433 | 1.84 | 1.19 |
|  | 9435 | 2.27 | 5 |
|  | 9520 | 2.46 | 1.61 |
|  | 9530 | 2.93 | 1.84 |
|  | 9600 | 2.48 | 1.62 |
|  | 9611 | 2.71 | 5.75 |
|  | 9921 | 2.04 | 1.47 |
